# The Atlanta Medical Society

**Published:** 1883-05

**Authors:** 


### Societies.

### THE ATLANTA MEDICAL SOCIETY.

*Reported for the Atlanta Medical Register.*

ATLANTA, March, 1883.

The President, Dr. W. D. Bizzell, in the chair.

Dr. James B. Baird said that acute tonsilitis had been very prevalent of late, perhaps unusually so, and desired to know the opinion of members as to the most satisfactory treatment. He expressed the opinion that laxatives and quinine were generally indicated, with, occasionally, an anodyne. Gargles of a saturated solution of chlorate of potash in glycerine and water, with or without an astringent, as the tincture of chloride of iron or sulphate of zinc, are comfortable and useful. Is confident that the application of lunar caustic is decidedly serviceable. When the swelling is considerable a poultice externally applied is soothing; so is scarification of the tonsils under the same circumstances. Of course, if abscesses form they should be promptly opened, thereby averting days of suffering.

He understands perfectly well that in chronic enlargement of the tonsils it sometimes becomes desirable, or even necessary, to remove them, but he believes the practice is being run into the ground on the "one idea" system. The operation, no more than circumcision, should be needlessly performed.

Dr. O'Brien remarked that in acute tonsilitis he relied mainly upon nitrate of silver solution, 60 grs. to the ounce of water applied to the tonsils. He had recently succeeded in aborting an invasion of the disease by this remedy. What greatly confirmed his confidence in the remedy was the fact that in a second attack of the same disease in the same patient, the solution had been used with a like favorable result. He had used, however, at the same time, internally, ten gr. doses of sulpho-carbolate of soda; adding that he had, also, found the same medicine valuable in diphtheria. Salt and water gargles he likewise recommended as an excellent local remedy.

Dr. W. D. Bizzell mentioned the significant fact that professional singers decline the application of nitrate of silver to the tonsils and throat, while readily submitting to the excision of the glands by the knife.

Dr. Connally agreed with Dr. O'Brien in the use of nitrate of silver in tonsilitis, but he had used it in the chronic form of the disease—40 grs. to the ounce of water, applied on a probang to the diseased parts, once a day, or once in two days, if the local irritation from the salt was too great.

Dr. G. H. Noble spoke in favor of Lugol's solution of iodine in chronic tonsilitis. ʒjj of the crystals of iodine to the ℥ of water would also be often an effectual remedy.

Dr. J. H. Logan would add to Dr. Noble's treatment in chronic tonsilitis with hypertrophy the injection of the tinc. of iodine into the substance of the glands—an ordinary hypodermic syringe, with a needle sufficiently long, being the only instrument necessary for the operation.

